# The intrapleural volume threshold for ultrasound detection of pneumothoraces: An experimental study on porcine models

**DOI:** 10.1186/1757-7241-21-11

**Published:** 2013-03-01

**Authors:** Nils Petter Oveland, Eldar Søreide, Hans Morten Lossius, Frode Johannessen, Kristian Borup Wemmelund, Rasmus Aagaard, Erik Sloth

**Affiliations:** 1 Department of Research and Development, Norwegian Air Ambulance Foundation, Mailbox 94, Droebak, 1441, Norway; 2 Department of Anesthesiology and Intensive Care, Stavanger University Hospital, Stavanger, Norway; 3 Department of Surgical Sciences, University of Bergen, Bergen, Norway; 4 Department of Radiology, Stavanger University Hospital, Stavanger, Norway; 5 Faculty of Health Sciences, Institute of Clinical Medicine, Aarhus University, Aarhus, Denmark; 6 Department of Anesthesiology, Regional Hospital of Randers, Randers, Denmark; 7 Department of Anesthesiology and Intensive Care, Aarhus University Hospital, Aarhus, Denmark

**Keywords:** (MeSH terms): Pneumothorax, Ultrasonography, Chest x-ray, Computed tomography, Pleura and (models animal)

## Abstract

**Background:**

Small pneumothoraxes (PTXs) may not impart an immediate threat to trauma patients after chest injuries. However, the amount of pleural air may increase and become a concern for patients who require positive pressure ventilation or air ambulance transport. Lung ultrasonography (US) is a reliable tool in finding intrapleural air, but the performance characteristics regarding the detection of small PTXs need to be defined. The study aimed to define the volume threshold of intrapleural air when PTXs are accurately diagnosed with US and compare this volume with that for chest x-ray (CXR).

**Methods:**

Air was insufflated into a unilateral pleural catheter in seven incremental steps (10, 25, 50, 100, 200, 350 and 500 mL) in 20 intubated porcine models, followed by a diagnostic evaluation with US and a supine anteroposterior CXR. The sonographers continued the US scanning until the PTXs could be ruled in, based on the pathognomonic US “lung point” sign. The corresponding threshold volume was noted. A senior radiologist interpreted the CXR images.

**Results:**

The mean threshold volume to confirm the diagnosis of PTX using US was 18 mL (standard deviation of 13 mL). Sixty-five percent of the PTXs were already diagnosed at 10 mL of intrapleural air; 25%, at 25 mL; and the last 10%, at 50 mL. At an air volume of 50 mL, the radiologist only identified four out of 20 PTXs in the CXR pictures; i.e., a sensitivity of 20% (95% CI: 7%, 44%). The sensitivity of CXR increased as a function of volume but leveled off at 67%, leaving one-third (1/3) of the PTXs unidentified after 500 mL of insufflated air.

**Conclusion:**

Lung US is very accurate in diagnosing even small amounts of intrapleural air and should be performed by clinicians treating chest trauma patients when PTX is among the differential diagnoses.

## Background

Pneumothorax (PTX), defined as the presence of air in the pleural space [1], is a dynamic condition with a wide continuum of severity. Tension PTXs can occur when air is allowed to enter the pleural space from the lung parenchyma, mediastinum or through the chest wall without exiting. The increased pressure may quickly lead to a collapse of the lung and cardiovascular compromise [2]. Failure to diagnose and treat this condition may cause patient death [3]. Smaller PTXs are often more subtle and may be managed without insertion of chest tubes [4,5]. Still, they may expand and become of concern in patients who require positive pressure ventilation [6] or are subject to air ambulance transportation with limited monitoring capacity and air pressure changes [7]. Progression of a PTX is a strong individual predictor for failure when using an observational strategy, with a 70-fold increased risk for needing a tube thoracostomy [6]. As a notable cause of preventable death [8], a prompt and accurate test to detect PTX is warranted [9]. The diagnostic challenge is that the physical examination of the patient, including auscultation, is often insufficient [4,10] and that supine anteroposterior chest x-rays (CXRs) frequently fail to detect any intrapleural air [11,12]. These occult PTXs may subsequently be found on computed tomography scans, but the involved time delay and radiation hazard are potential limitations [13,14]. Lung ultrasonography (US) is a non-invasive, non-radiating, rapid and repeatable bedside diagnostic test for detecting PTX that has been shown to be more sensitive and equally specific as supine CXR [15]. We know that US is highly accurate at detecting any progression of PTX size [16], but we lack a clear understanding of the exact amount of intrapleural air needed for US imaging of PTXs to be successful. The aim of our study was to define the threshold volume at which we could diagnose PTXs with US and to compare this to CXR.

## Materials and methods

### Study design and setting

This was a laboratory study using a porcine PTX model [16,17] at the vivarium at the Institute of Clinical Medicine, Aarhus University, Denmark. Qualified and experienced animal caretaker personnel monitored the health of the animals during the study period. The experiments complied with the guidelines for animal experimental studies issued by the Danish Inspectorate for Animal Experimentation under the Danish Ministry of Justice, which also approved the study. The study adhered to the principles in the Guide for the Care and Use of Laboratory Animals [18]. Twenty pigs with a mean body weight of 54.1 kg (standard deviation of 4.9 kg) were used. To create an experimental PTX in each porcine model, air was introduced through a small chest tube and the intrapleural volume was gradually increased through seven incremental series of insufflation, followed by a diagnostic evaluation with US and a supine anteroposterior CXR. By drawing envelopes with a right or left code, eight pigs were randomized to have a chest drain on the right thoracic side; and 12, the left side. No pigs had bilateral PTX.

### Animal model

**Figure 1:**
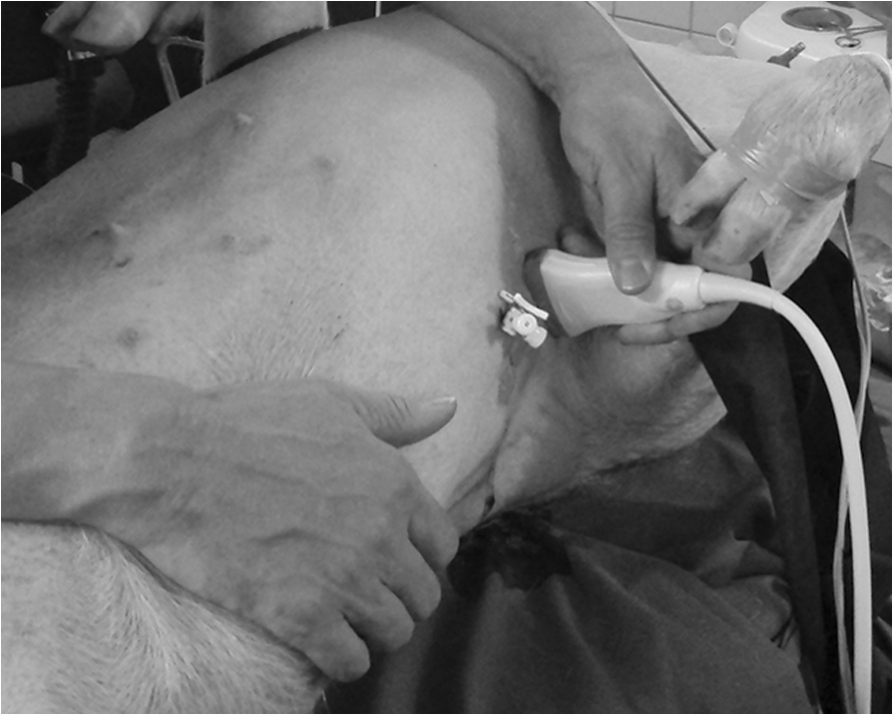
**Ultrasound scanning of the porcine pneumothorax model.** The catheter enters the pleural space and is used for incremental air insufflations.

The preparation of the porcine PTX model has been described in detail in previous studies [16,17]. In brief, the pigs were anesthetized, intubated and fixated in the supine position on the operating table. A small thoracotomy was performed at the crossing of the fifth to seventh intercostals with the anterior axillary line, and a three-way stopcock catheter (BD Connecta, BD Medical, Franklin Lakes, NJ, USA) was inserted into the pleural space (Figure 1). The catheter was then anchored to the surrounding muscle and fascia. Excessive air introduced by the catheter placement was withdrawn using a 10 mL syringe. To control that the pleural space was empty, the study supervisor scanned the thorax for presence of intrapleural air. The respirator (GE S5 Advance Carestation™, Datex-Ohmeda, GE Healthcare, London, UK) was adjusted to a tidal volume of 11 to 15 mL/kg, a respiratory rate of 10 to 12 breaths/minute, a positive end expiratory pressure of two to four cm H_2_O and an inspiratory oxygen fraction of 30%. The end-tidal carbon dioxide level was kept within the normal range (4.0 to 6.5 kPa). All animals were monitored by electrocardiography, and their core temperatures, invasive arterial blood pressures, oxygen saturations and end-tidal carbon dioxide levels were trended. At the conclusion of data collection, each animal was euthanized with an injection of pentobarbital.

### Diagnostic tests

Examinations with lung US and supine anteroposterior CXR were performed to exclude any lung pathology after the catheter insertion and again after each of the seven consecutive series of air insufflations (10, 25, 50, 100, 200, 350 and 500 mL of air).

#### US

**Figure 2:**
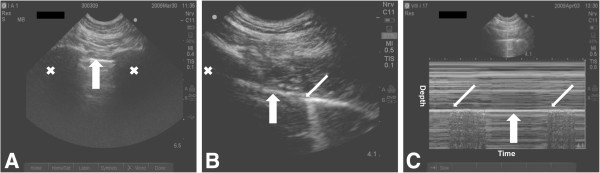
**Ultrasound detection of pneumothorax.** Symbols: Wide arrow, pleural line; thin arrow, lung point; X, rib shadow. **A**) The pleural line between two ribs is identified close to the sternum. **B**) The probe is rotated to align in the intercostal space and then gradually moved towards the lateral-posterior area of the chest, always following the pleural line. The aim is to detect “to and fro” horizontal movement at the pleural line, which is called lung sliding, excluding air between the pleural layers (visible only on video clips). Even with a pneumothorax present, the lung is still in contact with the interior chest wall. This contact point is called the “lung point” and moves in and out of the ultrasound screen alongside the pleural line, synchronous with respiration (visible only on video clips). The lung point is 100% pathognomonic for pneumothorax. **C**) In time-motion mode, the lung point appears as two distinct sonographic patterns; one pattern is suggestive of normal lung sliding (seashore pattern) and is interposed by horizontal lines (stratosphere pattern) that are seen when intrapleural air is present.

Two anesthesiologists with moderate experience (i.e. less than one year of clinical practice with lung US) performed the US scanning of the 20 lungs in each volume series (n = 7) with a set time limit of three minutes per lung. The first sonographer scanned the first 12 porcine models using a M-Turbo (SonoSite Inc., Bothell, WA, USA) with a C11 micro convex 8–5 MHz broadband array transducer (SonoSite Inc.) and the second sonographer the last eight models using Vivid Q (GE Healthcare, Horten, Norway) with a 12 L-RS multifrequency 6–13 MHz linear array transducer (GE Healthcare, Horten, Norway). The study design precluded the sonographers from being blinded to the thoracic side with the chest drain, but the diagnostic criteria of PTX were very strict based on the demonstration of the dynamic US sign “lung point” [19] in both brightness mode and time-motion mode. The scanning followed a stepwise technique to identify specific US signs as illustrated in Figure 2 and further embellished here: 1) The bat sign: On a longitudinal scan at the anterior chest two ribs with the pleural line in-between are identified. 2) Detection of lung sliding and/or B-lines: The probe is rotated approximately 90 degrees to align in the intercostal space and then gradually moved towards the lateral-posterior parts of the chest. The aim is to detect the presence or absence of horizontal movements of the pleural layers, called lung sliding and/or vertical B-lines (i.e. reverberation artifact originating from the pleural line). Lung sliding and/or B-lines rule out PTX, while both signs are absent if the pleural layers are separated by air. 3) Sea shore sign in time-motion mode: When lung sliding is present the US image has a granular appearance under the pleural line (resembling sand) and horizontal lines above the pleural line (resembling the horizon). 4) Stratosphere sign in time motion-mode: Straight horizontal lines throughout the image indicate abolished lung sliding. 5) Lung point indicating PTX: The boarder between the intrapleural air and the part of the lung still in contact with the interior chest wall is called the lung point. It appears on the US screen as two distinct sonographic patterns of lung sliding and no-lung sliding that interchange synchronous with respiration, and can be displayed in both brightness and time-motion mode.

Once the sonographers confirmed the diagnosis of PTX, the scanning ceased and the corresponding intrapleural threshold volume was noted.

#### CXR

**Figure 3:**
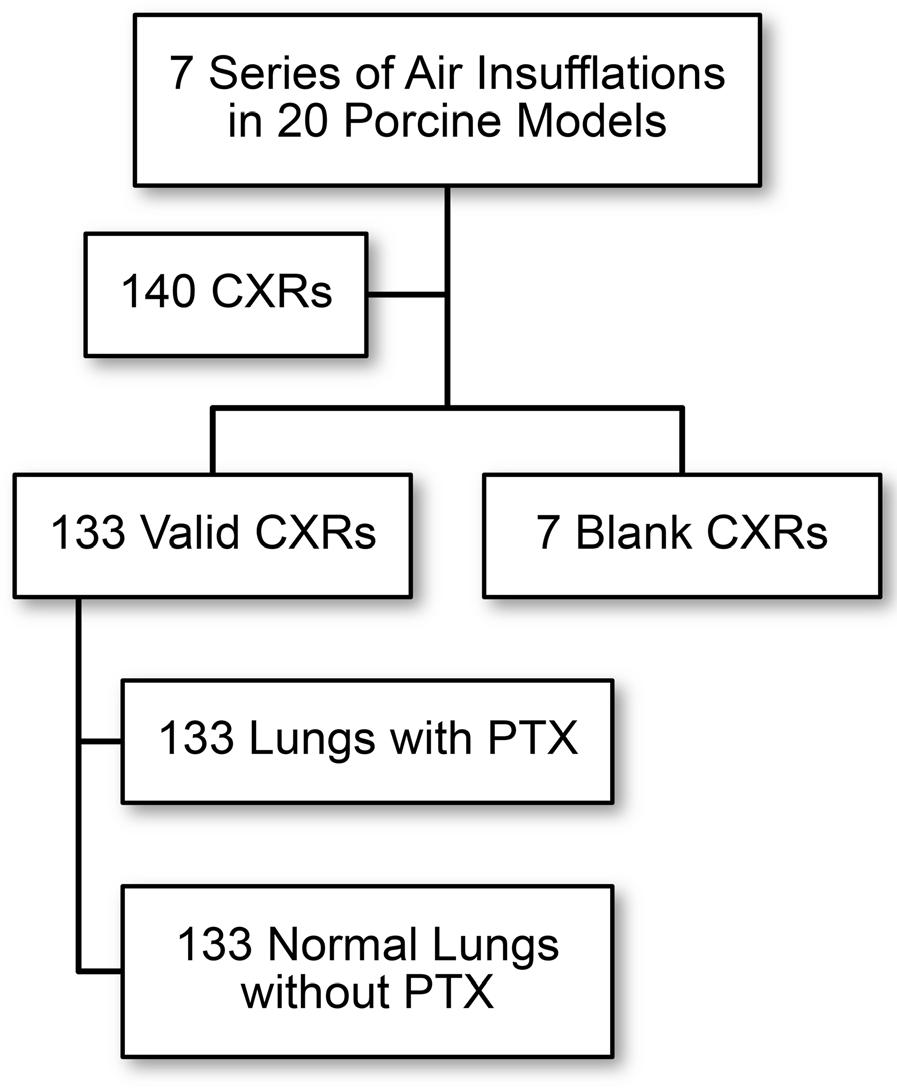
Flow chart of the number of lungs analyzed for pneumothorax by the radiologist.

**Figure 4:**
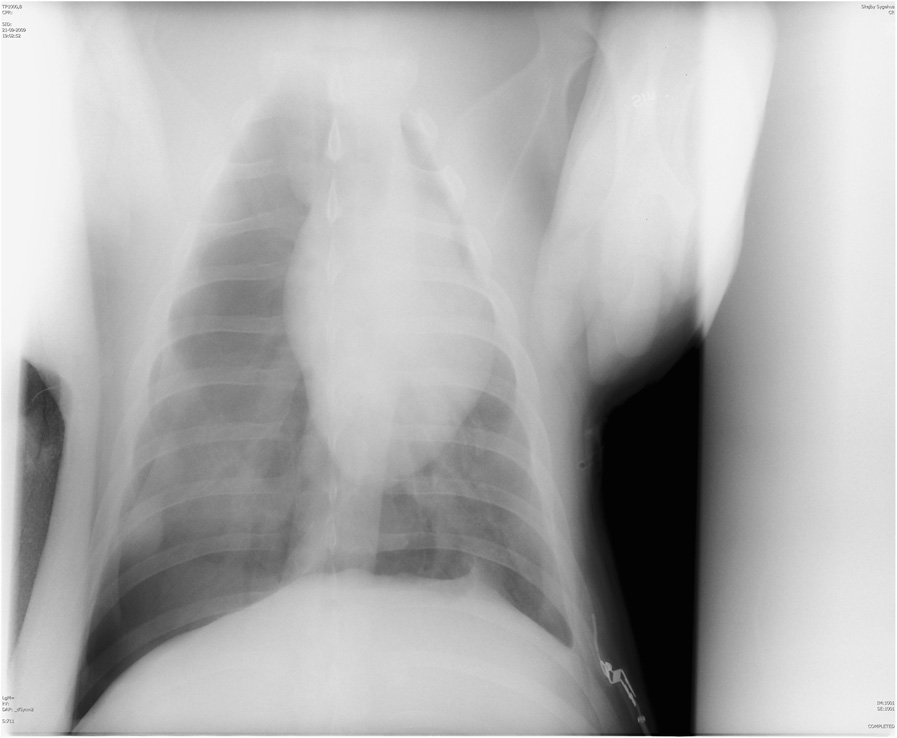
**A supine anteroposterior chest x-ray of one porcine model diagnosed with a basal right-sided pneumothorax.** Although the chest anatomy between pigs and humans differ, the intrapleural distribution of air is similar with air collecting in the anteromedial and basal recesses. The radiological review of pneumothorax for humans was therefore also applied on our models. The radiologist interpreted each picture for a readily apparent visceral pleural line without distal lung markings, depressed diaphragm and the deep sulcus sign (i.e. enlargement of the costophrenic angle).

The CXRs were given encrypted names and presented to a senior radiologist in a sequence determined by a randomization procedure that assigned numbers to the pictures. The radiologist had to determine the presence or absence of a PTX on the right and left thoracic sides in each CXR (Figure 3). He was told that the porcine models could have a unilateral PTX, bilateral PTXs or even two normal lungs; additionally, the chest drains that we used were radiologically transparent to conceal any diagnostic information. The radiological definition of PTX is illustrated in Figure 4[20]. The CXRs were viewed using a DICOM viewer (Phillips R 2.6, Philips Medical Systems, Amsterdam, the Netherlands), and the PTX diagnoses were scored on a five-point Likert scale (1 = definitely absent, 2 = probably absent, 3 = possibly present, 4 = probably present and 5 = definitely present), with a score of four or five being considered positive for PTX [21].

### Data analysis

The main outcome measure in this study was to define the threshold at which PTXs become detectable using US imaging. The mean threshold volumes for the 20 pigs were presented as absolute and mean values (mL) with standard deviations. Furthermore, we calculated the sensitivity of both CXR and US for diagnosing PTX at increasing volumes of insufflated air by dividing the number of true positives found by the diagnostic tests with the actual number of PTXs in the porcine models. An online calculator was used to calculate the confidence intervals (http://vassarstats.net; Vassar College, Poughkeepsie, NY, USA), and the other analyses were performed using SPSS V.18.0 (IBM SPSS, Armonk, NY, USA).

## Results

**Figure 5:**
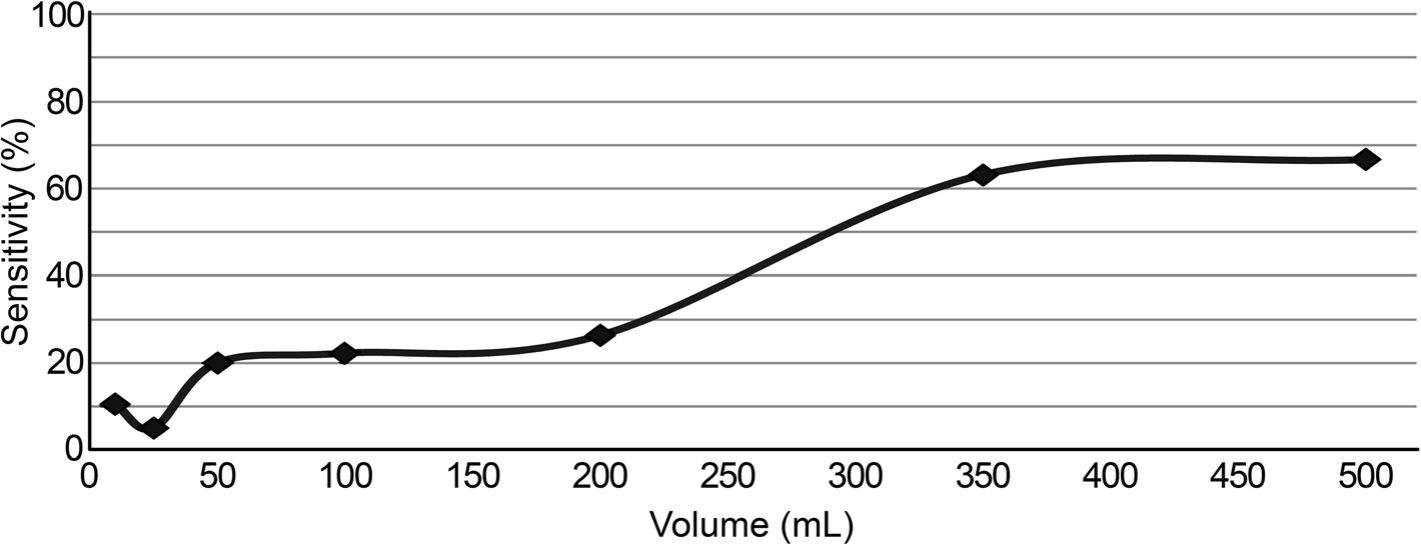
The sensitivity of chest x-ray for diagnosing pneumothorax as a function of increasing intrapleural air volume.

The total mean threshold volume to confirm the diagnosis of PTX in 20 porcine models using US was 18 mL (standard deviation [SD] of 13 mL). The sonographers detected 13 PTXs (65%) at an intrapleural volume of only 10 mL; an additional five PTXs (25%) were detected at 25 mL, and the last two PTXs (10%) were identified at 50 mL. The characteristics and threshold volumes of the individual animals are illustrated in Table 1. The absolute difference in mean threshold volume between the sonographer using the microconvex probe and the sonographer using the linear probe was 8 mL (i.e. a mean threshold detection volume of 15 mL [SD 12 mL] and 23 mL [SD 13 mL] respectively). The sensitivity of US detection of PTX increased from 65% to 90% and 100% as the intrapleural air volume increased from 10 mL to 25 mL and 50 mL, respectively (Table 2). At an air volume of 50 mL, the sonographers diagnosed all PTXs, while the radiologist only identified four out of 20 PTXs on the CXR pictures; i.e., a sensitivity of 20% (95% CI: 7%, 44%). As shown in Figure 5, the sensitivity of CXR as a function of volume gradually increased. However, the sensitivity leveled off at volumes ≥ 350 mL and was 67% at the maximum volume of 500 mL (95% CI: 41%, 86%), leaving one-third (1/3) of the PTXs unidentified. The diagnostic performance of supine anteroposterior CXR in diagnosing PTX is summarized in Table 3.

**Table 1:** Threshold volume of intrapleural air for ultrasound confirmation of pneumothorax in 20 porcine models

**Pig**	**Weight (Kg)**	**Catheter level (Costa)**	**Thoracic side (Pneumothorax)**	**Threshold volume (mL)**^***a***^
1	61.0	3–4	Left	10
2	58.5	3–4	Left	10
3	55.5	5–6	Right	25
4	58.3	4–5	Left	10
5	58.0	5–6	Right	10
6	58.0	5–6	Left	10
7	58.0	4–5	Left	10
8	59.5	4–5	Left	10
9	58.0	5–6	Right	10
10	61.0	5–6	Left	10
11	46.5	5–6	Right	10
12	50.3	5–6	Right	50
13	52.4	6–7	Right	25
14	49.6	5–6	Left	25
15	48.6	5–6	Left	10
16	50.0	6–7	Left	50
17	52.0	5–6	Left	25
18	50.0	5–6	Right	10
19	46.4	6–7	Left	10
20	50.1	6–7	Right	25

^*a*^ The insufflated air volume at which the sonographer diagnosed pneumothorax by identifying the specific ultrasound “lung point” sign.

**Table 2:** The sensitivity of ultrasound detection of pneumothorax at different intrapleural air volumes

**PTX volume**	**TP**	**n**	**Sensitivity (%)**	**95% CI**
10 mL	13	20	65	(41, 84)
25 mL	18	20	90	(67, 98)
50 mL	20	20	100	(80, 100)

*Abbreviations:* PTX, pneumothorax; TP, true positive; n, number of lungs with PTX.

**Table 3:** The sensitivity of anteroposterior supine chest x-ray in diagnosing pneumothorax at different intrapleural air volumes

**PTX volume**	**TP**	**n **^***a***^	**Sensitivity (%)**	**95% CI**
10 mL	2	19	11	(2, 35)
25 mL	1	20	5	(0, 27)
50 mL	4	20	20	(7, 44)
100 mL	4	18	22	(7, 48)
200 mL	5	19	26	(10, 51)
350 mL	12	19	63	(39, 83)
500 mL	12	18	67	(41, 86)

*Abbreviations:* PTX, pneumothorax; TP, true positive; n, number of lungs with PTX.

^*a*^ Due to some blank chest x-ray pictures, the “n” values differ in the volume series.

## Discussion

This animal study demonstrates that US has the capacity to diagnose very small PTXs. The mean threshold volume of 18 mL of intrapleural air is exceptionally low and approximates what is possible to detect using the reference standard computed tomography. Furthermore, the results confirm that supine anteroposterior CXR is a poor diagnostic technique to detect air within the chest cavity in pigs. Similarly, radiographic studies using human cadavers in the supine position have demonstrated that up to 400 mL of air in the pleural space is required to detect PTXs [22]. In our study, even 500 mL of air was insufficient, as the radiologist only diagnosed 12 out of 18 (67%) of these large PTXs. Other studies using US on porcine models with PTX show that an average of 4 mL/kg [23] or 50 mL [24] was required to obliterate the normal sliding between the pleural layers. Our study is the first to present the exact volume threshold of US detection of PTX based on not only obliteration of normal lung sliding between the pleural layers but also identification of the “lung point”: the only PTX-specific US sign [19].

Competing concerns, such as spinal injury, hemodynamic compromise and the need for clinical interventions, often confine trauma patients to the supine position, where the intrapleural air collects anteromedially to the lung parenchyma. Air in this location is particularly difficult to detect and quantify in supine CXR [25]. In all of the porcine models, the sonographers observed that small PTXs resided close to the sternum on the anterior chest. This is the first lung area to be examined in the eFAST protocol [26], which may explain the increased sensitivity of US to diagnose PTXs that are otherwise undetected on CXRs. Still, there is a scientific debate regarding whether US should replace CXR [27]. The British Thoracic Society maintains their caution to use US in the detection and management of PTXs, reflected in the last 2010 British Thoracic Society guidelines on pleural procedures and thoracic US [28]. The World Interactive Network Focused on Critical Ultrasound and the International Liaison Committee on Lung Ultrasound have expressed their concern in their comments to the guidelines [27]; in March 2012, they published the first international evidence-based recommendations for point-of-care lung US [29]. In the paper, US is presented as the preferred diagnostic test in the initial evaluation of critically ill or injured patients with suspected PTX. This conflict between the guidelines calls for further research. The British Thoracic Society made a statement that “if the PTX is so small as to be undetectable on CXR, then it is unlikely to require intervention and use of US will not have changed the management” [27], which may be valid for spontaneously breathing patients but not in patients receiving positive pressure ventilation. Detecting even small amounts of air is highly relevant, as mechanical ventilation triples the risk of observation failure and the need for a chest tube [6]. The British Thoracic Society acknowledges this clinical challenge [27] but questions whether small PTXs may fail to be detected by US [30]. Our study strengthens the evidence to the contrary, demonstrating that US could be the preferred method for diagnosing these miniscule PTXs. The results indicate that US outperform supine CXR and are significantly more reliable in the detection of small- and medium-sized PTXs. This finding is evident when comparing sensitivities between the two diagnostic tests from the first three volume series (10 mL, 25 mL and 50 mL), where a PTX volume of 50 mL was enough for US imaging to identify all PTXs in porcine models but CXR missed 80%.

Porcine anatomy is not identical to human anatomy; however, their respiratory and cardiovascular systems are similar [31,32]. Therefore, before commencing this study, we validated and tested our experimental porcine model against computed tomography and noted that the PTX topography was similar to what is expected in human trauma patients. Furthermore, our studies also revealed that the PTX volume could easily be altered through insufflation and deflation [16,17]. In fact, the design of this study, with incremental injections of air combined with the radiation hazard posed by serial CXR or computed tomography scans, precludes using this experimental approach in human subjects. However, there are limitations. First, the thoracotomies may have introduced small amounts of air into the pleural cavities when the catheters were introduced. This excessive air was withdrawn using a syringe, but some residual air may have been added to the insufflated air volumes specified in the study protocol. The results reported in this study (i.e. the mean volume threshold in mL) may be too low because of this. Second, lung US is a very operator-dependent examination, and the sonographers had up to one-year experience; the reproducibility of the threshold volume is uncertain in more inexperienced hands and real clinical settings. Third, two ultrasound devices and probes (i.e. one microconvex and one linear) were used, with potentially different characteristics and ability to detect PTX. The sub-analysis based on the probes showed an absolute difference in threshold detection volume of only 8 mL, not found to be clinical relevant. Multiple transducers can be used to detect PTX, and the two probes used to scan in this study proved satisfactory. Finally, unlike the radiologist, the sonographers were not blinded to the thoracic side with the chest drain. This aspect of the procedure could have affected the US scan results and resulted in a lower threshold volume necessary to confirm the PTX diagnosis compared to blinded examinations. Still, in this study the diagnostic criteria for PTX was based on identification of the pathognomonic lung point sign in both brightness and time-motion mode. We believe the clear difference in accuracy between lung US and supine CXR in detecting intrapleural air that was determined in our study is not readily explained by this limitation.

## Conclusions

The mean threshold volume to confirm the diagnosis of PTX using lung US in a porcine model was only 18 mL, a result previously unreported. At 50 mL of intrapleural air, all PTXs were diagnosed using US while supine CXRs missed 80%. Therefore, lung US is a safe and accurate diagnostic test to diagnose even small PTXs that are otherwise undetectable and should be performed by clinicians treating chest trauma patients as an important adjunct to the clinical assessment.

## Abbreviations

CXR: Chest x-ray; PTX: Pneumothorax; SD: Standard deviation; US: Ultrasound

## Competing interests

The authors declare that they have no competing interests.

## Authors’ contributions

NPO, MD – Literature Search, Study Design, Data Collection, Data Interpretation, Writing, Critical Review of the manuscript, ES, MD, PhD – Data Analysis, Data Interpretation, Writing, Critical Review of the manuscript, HML, MD, PhD – Study Design, Writing, Critical Review of the manuscript, FJ, MD – Data Interpretation, Writing, Critical Review of the manuscript, KW, cand.med – Study Design, Data Analysis, Data Interpretation, Writing, Critical Review of the manuscript, RA, MD - Study Design, Data Analysis, Data Interpretation, Writing, Critical Review of the manuscript, ES, MD, PhD, DMsc – Literature Search, Study Design, Data Collection, Data Analysis, Data Interpretation, Writing, Critical Review of the manuscript. All authors read and approved the final manuscript.
